# Counter-Irritation

**Published:** 1882-11

**Authors:** 


### Counter-Irritation.

Of all therapeutic measures, there is probably no single one so universal, or so potent in its application as counter-irritation. The disputed point as to whether it produces its results through the agency of the nervous or the circulatory sytsem, has no practical interest for the busy physician. He has but little time for theories or physiological experimentation, what he most desires are facts and results. In counter-irritation he has them both; it *can* cure, and it *does* cure. It matters but little in kind, but greatly in degree, what counter-irritant is used. When we desire a profound impression, as in the initial stage of asthenic pleurisy, nothing short of a fly blister, and a big one at that, one that will cover the whole affected side, will do any good. A mustard plaster, under such circumstances, will be like a child playing at war, as compared with real battle. Whereas, when we meet an asthenic type of the same disease in a weakly and debilitated individual, the violent fly blister would be too powerful, and resort must be had to the gentler mustard. Much undeserved odium has been cast upon counter-irritation, as upon many other therapeutic measures, because of inferiority in quality of the article used. We have used half an ounce of cantharidal collodion with the result of merely soiling the part to which it was applied, it did not even *redden* the skin, while half a drachm of a good article, applied to the same part, on the same person, blistered most terribly.

Many object to counter-irritation of a severe character, on the ground that it produces an external sore that is sometimes very hard to heal. That this is true no one can doubt. It does sometimes happen that very tedious and painful abrasions of the skin, or sores, do result from fly blisters or tincture of iodine, or some other equally powerful counter-irritant. But these are the exceptions and ought not to militate against the rule, which is, that this form of treatment is an exceedingly valuable one in nearly all affections of an inflammatory nature. A large volume could be written on the different counter-irritants, and the great value of counter-irritation, but it is not our purpose to do more than merely call your attention to the matter. Every physician must wisely judge what cases are suitable for this form of treatment and what articles are appropriate to the effect desired. But remember, that while it will be all very well to resort to internal medication, counter-irritation *can do no serious* harm, and it will oftentimes amazingly aid the action of your drugs.

